# BNT162b2-elicited neutralization of Delta plus, Lambda, Mu, B.1.1.519, and Theta SARS-CoV-2 variants

**DOI:** 10.1038/s41541-022-00462-4

**Published:** 2022-04-08

**Authors:** Jianying Liu, Yang Liu, Hongjie Xia, Jing Zou, Scott C. Weaver, Kena A. Swanson, Hui Cai, Mark Cutler, David Cooper, Alexander Muik, Kathrin U. Jansen, Ugur Sahin, Xuping Xie, Philip R. Dormitzer, Pei-Yong Shi

**Affiliations:** 1 Department of Microbiology and Immunology, University of Texas Medical Branch, Galveston, TX USA; 2 Institute for Human Infections and Immunity, University of Texas Medical Branch, Galveston, TX USA; 3 Department of Biochemistry and Molecular Biology, University of Texas Medical Branch, Galveston, TX USA; 4 Institute for Translational Sciences, University of Texas Medical Branch, Galveston, TX USA; 5 Center for Biodefense & Emerging Infectious Diseases, University of Texas Medical Branch, Galveston, TX USA; 6 Sealy Institute for Vaccine Sciences, University of Texas Medical Branch, Galveston, TX USA; 7 Pfizer Vaccine Research and Development, Pearl River, NY USA; 8 BioNTech, Mainz, Germany

**Keywords:** RNA vaccines, SARS-CoV-2

## Abstract

BNT162b2-elicited human sera neutralize the currently dominant Delta SARS-CoV-2 variant. Here, we report the ability of 20 human sera, drawn 2 or 4 weeks after two doses of BNT162b2, to neutralize USA-WA1/2020 SARS-CoV-2 bearing variant spikes from Delta plus (Delta-AY.1, Delta-AY.2), Delta-∆144 (Delta with the Y144 deletion of the Alpha variant), Lambda, B.1.1.519, Theta, and Mu lineage viruses. Geometric mean plaque reduction neutralization titers against Delta-AY.1, Delta-AY.2, and Mu viruses are slightly lower than against USA-WA1/2020, but all sera neutralize the variant viruses to titers of ≥80, and neutralization titers against the Delta-∆144, Lambda, B.1.1.519 and Theta variants not significantly reduced relative to those against USA-WA1/2020. The susceptibility of Delta plus, Lambda, B.1.1.519, Theta, Mu, and other variants to neutralization by the sera indicates that antigenic change has not led to virus escape from vaccine-elicited neutralizing antibodies and supports ongoing mass immunization with BNT162b2 to control the variants and to minimize the emergence of new variants.

## Introduction

As of October 25, 2021, severe acute respiratory syndrome coronavirus 2 (SARS-CoV-2) has caused over 243 million infections and more than 4.9 million deaths due to coronavirus disease 2019 (COVID-19; https://coronavirus.jhu.edu/). Since its emergence in late 2019, SARS-CoV-2 has accumulated mutations, leading to variants with higher transmission, more efficient replication, and potentially immune evasion^1–5^. Many of these mutations have occurred in the viral spike glycoprotein, which is responsible for binding to the host receptor, angiotensin-converting enzyme 2, during virus entry. Based on the effects of mutations on viral transmission, disease severity, and clinical diagnosis, the World Health Organization (WHO) has categorized SARS-CoV-2 strains into “variants of concern (VOC),” “variants of interest (VOI),” and “variants under monitoring (VUM)” (https://www.who.int/en/activities/tracking-SARS-CoV-2-variants/). As of the submission of this study in September 2021, VOC include Alpha (B.1.1.7), Beta (B.1.351), Gamma (P.1), and Delta (B.1.617.2); VOI include Lambda (C.37) and Mu (B.1.621). As the pandemic continues, it is critical to monitor closely the new variants for their transmission, pathogenesis, and potential escape from vaccines and therapeutics.

BNT162b2 is an mRNA vaccine expressing the full-length prefusion spike glycoprotein of SARS-CoV-2, stabilized in the prefusion conformation^6^. BNT162b2 has recently been approved for vaccination of individuals 16 years of age and older and has been authorized under emergency use provisions for immunization of those 5–15 years old by the US Food and Drug Administration. Although BNT162b2 mRNA encodes the original spike protein from the Wuhan isolate^7^, the sera of those immunized with BNT162b2 can neutralize all tested variants, including the currently circulating Delta variant^2,8–13^. However, some variants are less efficiently neutralized than others, with the Beta and Kappa variants showing the greatest decrease to date^8–10^. The explosive recent spread of the Delta variant to 119 countries and its association with breakthrough infections in vaccinated people prompted us to examine the closely related Delta plus variants, such as (i) Delta-AY.1 (first detected in India and spread to 52 countries, including the USA); (ii) Delta-AY.2 (first detected in the USA and spread to 11 countries); and (iii) Delta-∆144 (first detected in Vietnam and spread to 17 countries) (https://www.gisaid.org/hcov19-variants/). In addition to the Delta variants, the Lambda variant (C.37; first detected in Peru) has spread to 46 countries with high prevalence in South America; the Theta variant (P.3; first identified in the Philippines) was considered as a VOI from February to July of 2021; the Mu variant (B.1.621; first documented in Colombia) has been found in 61 countries; the B.1.1.519 variant has emerged and became dominant in Mexico during the first months of 2021 (https://www.gisaid.org/hcov19-variants/). Consequently, the WHO has designated the Delta sublineages, Delta-AY.1 and Delta-AY.2, as VOC, Lambda and Mu as VOI, and B.1.1.519 as VUM. Here, we report BNT162b2 vaccine-elicited neutralization against these new variants.

## Results

### BNT162b2-elicited neutralization of SARS-CoV-2 variants

We aimed to study the impact of antigenic variation in the SARS-CoV-2 spike glycoprotein on neutralization by antibodies elicited by the wild type (WT) spike glycoprotein encoded by BNT162b2 RNA. Therefore, we used a reverse genetic system to generate a panel of SARS-CoV-2 with a USA-WA1/2020 genetic background (a viral strain isolated in January 2020 and defined as WT) and spike glycoproteins from the newly emerged variants (Supplementary Fig. 1a). Seven chimeric SARS-CoV-2s were prepared: (i) Delta-AY.1-spike with T19R, T95I, G142D, E156G, F157/R158 deletion (∆157/158), W258L, K417N, L452R, T478K, K558N, D614G, P681R, and D950N mutations (GISAID accession ID: EPI_ISL_2676768); (ii) Delta-AY.2-spike with T19R, V70F, G142D, E156G, ∆157/158, A222V, K417N, L452R, T478K, D614G, P681R, D950N, and V1228L (GISAID accession ID: EPI_ISL_2527809); (iii) Delta-∆144-spike (a Delta variant that has acquired a Y144-deletion from the Alpha variant) with T19R, G142D, ∆144, E156G, ∆157/158, A222V, L452R, T478K, D614G, P681R, and D950N (GISAID accession ID: EPI_ISL_2373110); (iv) Lambda-spike with G75V, T76I, R246-G252 deletion (∆246-252), D253N, L452Q, F490S, D614G, and T859N (GISAID accession ID: EPI_ISL_1138413); (v) B.1.1.519-spike with T478K, D614G, P681H, and T732A (GISAID accession ID: EPI_ISL_876555); (vi) Theta-spike with L141-V143 deletion (∆141–143), A243-L244 deletion (∆243-244), Y265C, E484K, N501Y, D614G, P681H, E1092K, H1101Y, and V1176F (GISAID accession ID: EPI_ISL_1525595); and (vii) Mu-spike with T19R, T95I, insertion 143T, Y144S, Y145N, R346K, E484K, N501Y, D614G, P681H, D950N (GISAID accession ID: EPI_ISL_3430087). The WT and all seven chimeric viruses were rescued from infectious cDNA clones and titered by plaque assay on Vero E6 cells. Theta-spike and Mu-spike viruses developed smaller plaques than other chimeric variant viruses (Supplementary Figure 1b). All chimeric viruses had infectious titers of greater than 10^7^ plaque-forming units (PFU)/ml. The ratio of viral RNA to PFU was quantified for each virus; no significant differences in viral RNA-to-PFU ratios were detected between the WT and chimeric variant viruses (Supplementary Fig. 1c), indicating similar specific infectivities. Sequencing of viral stocks confirmed that there were no undesired mutations in the spike gene.

**Fig. 1:**
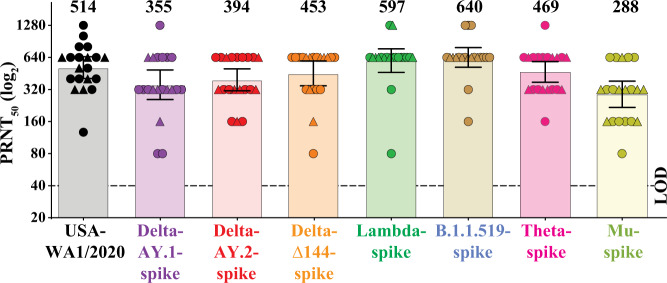
BNT162b2 vaccine-elicited neutralization of SARS-CoV-2 variants. The plot presents the PRNT_50_ titers of 20 human sera (drawn 2 or 4 weeks after two 30-µg doses of BNT162b2, spaced three weeks apart) against USA-WA1/2020 isolate and its chimeric viruses bearing distinct variant spikes. Serum samples obtained at 2 weeks or 4 weeks are represented by circles and triangles, respectively. Individual PRNT_50_ values are presented in Supplementary Table 1. Each data point represents the geometric mean PRNT_50_ against the indicated virus obtained with a serum specimen. The PRNT_50_ values against each variant were determined in duplicate assays of one experiment for each variant; the PRNT_50_ values for USA-WA1/2020 were pooled from three independent experiments; and the geometric means were calculated (*n* = 20). The values represent the geometric means with 95% confidence intervals. The numbers above the bars indicate geometric mean titers. The limit of detection (LOD) of the PRNT assay is 1:40 and indicated by a dashed line. Statistical analysis was performed using the two-tailed Wilcoxon matched-pairs signed-rank test. The statistical significances of the differences between geometric mean titers in the USA-WA1/2020 neutralization assay and in each variant virus neutralization assay with the same serum samples are as follows: *P* = 0.0253 for Delta-AY.1-spike; *P* = 0.0253 for Delta-AY.2-spike; *P* = 0.260 for Delta-∆144-spike; *P* = 0.156 for Lambda-spike*; P* = 0.018 for B.1.1.519-spike; *P* = 0.328 for Theta-spike; and *P* < 0.001 for Mu-spike.

We compared the neutralization susceptibility of the chimeric variant SARS-CoV-2s to a panel of 20 sera collected from BTN162b2-immunized human participants in the pivotal clinical trial^6,14^. As reported previously, the serum specimens were drawn 2 or 4 weeks after two immunizations with 30 μg of BNT162b2, spaced three weeks apart^11,12^. Each serum was tested simultaneously for its 50% plaque reduction neutralizing titers (PRNT_50_) against the WT and chimeric variant viruses (Supplementary Table 1). All the sera neutralized the WT and all the mutant viruses with titers of 1:80 or higher (Fig. 1). The geometric mean neutralizing titers against the WT, Delta-AY.1-spike, Delta-AY.2-spike, Delta-∆144-spike, Lambda-spike, B.1.1.519-spike, Theta-spike, and Mu-spike viruses were 520, 355, 394, 453, 597, 640, 469, and 288, respectively (Fig. 1). The neutralizing titers against the Mu variant were the lowest of the titers against any of the 7 viruses tested in this study. However, the reduction in titer against the Mu variant is not as great as the reduction in titer observed previously against the Beta variant^9^, and BNT162b2 protects against disease caused by Beta variant strains^8,15^. The neutralization of Delta plus variants Delta-AY.1 and Delta-AY.2, is only modestly reduced relative to neutralization of WT virus. We previously reported a similar neutralization result for another Delta plus strain, Delta-AY.3 (B.1.617.2.v2)^10^. Neutralization of the Delta-∆144, Lambda B.1.1.519, and Theta variants is not reduced relative to neutralization of WT virus. Overall, BNT162b2 immune sera efficiently neutralized all tested viruses.

## Discussion

We have taken a systematic approach to measuring BNT162b2-elicted neutralization of newly emerged variants. We use a reverse genetic system to generate chimeric SARS-CoV-2’s bearing spikes from distinct variants through site-directed mutagenesis or DNA synthesis. This approach has two major advantages: (i) it allows us to examine new variants as soon as their sequences become available, and (ii) it measures the impact of variant spikes on neutralizing activity without being affected by mutations outside the spike gene. Non-spike mutations are not directly relevant to the selection of vaccine spike sequences. We test all chimeric variants for neutralization by the same panel of 20 sera from BNT162b2-vaccinated trial participants, enabling us to make well controlled comparisons longitudinally to inform vaccine decision making^10–12,16^.

Our previous and current results suggest that BNT162b2-vaccinated sera neutralize the Delta and Delta plus variants more efficiently than they neutralize the Beta variant^10,11^. Real-world effectiveness of two doses of BNT162b2 against Beta variant-associated severe or fatal disease in Qatar and vaccine efficacy against Beta variant-associated COVID-19 in South Africa were both reported to have point estimates of 100%^8,15^. These results suggest that the observed breakthrough disease associated with the Delta variant is not due to antigenic change. The waning of neutralizing titers after two doses of vaccine appears to be a greater contributor to breakthrough COVID-19^15,17^. A third dose of BNT162b2 addresses the waning by eliciting neutralizing titers against wild type virus higher than those after the second dose as well as broader neutralization against the Delta and Beta variants^17^.

Recent studies using primary human airway cultures and a human lung epithelial cell line suggest that the Delta variant has improved replication fitness through mutation P681R-enhanced protease cleavage of the full-length spike to S1 and S2 subunits, as one mechanism leading to increased viral infection^18,19^. Viral RNA loads in the oropharynx from Delta variant-infected patients were over 1000-fold higher than those from the original Wuhan virus-infected individuals^20,21^. Collectively, the results suggest that improved viral fitness due to more efficient furin cleavage, rather than immune escape, may account for breakthrough infections of the Delta variant in vaccinated people. More efficient furin cleavage of the influenza fusion protein, hemagglutinin, increases viral pathogenicity to an even greater extent.

One limitation of this study is the potential for mutations to alter neutralization by affecting spike function rather than antigenicity (e.g., mutation P681R that improves viral replication through enhanced spike processing), despite the variant viruses exhibiting specific infectivities similar to that of the original USA-WA1/2020 virus on Vero E6 cells. Another limitation is that the study focuses only on the effect of spike glycoprotein mutations on neutralization in cell culture. Mutations outside the spike gene may also alter viral replication and/or host immune response.

The susceptibility of Delta, Delta plus, Lambda, Mu, and other variants to BNT162b2-elicited neutralization indicates that antigenic change does not yet appear to be the major mechanism of increased Delta variant pathogenicity or spread. This finding suggests that changing the strain of the spike glycoprotein encoded by the vaccine may not be the most effective response to the emergence and spread of the Delta variant. Nevertheless, Pfizer and BioNTech are preparing for the possibility that a strain change may someday be necessary by testing prototype, antigenically updated vaccines. The data in this paper support the ongoing BNT162b2 mass immunization strategy to control the current variants. High levels of coverage with effective vaccines may also minimize the emergence of new variants. Increasing the vaccination rate in the population, together with implementing public health measures, remains the primary means to end the COVID-19 pandemic.

## Methods

### Cells

Vero E6 cells, an African green monkey kidney epithelial cell line (ATCC, Manassas, VA, USA), were cultured in Dulbecco’s modified Eagle’s medium (DMEM; Gibco/Thermo Fisher, Waltham, MA, USA) with 10% fetal bovine serum (FBS; HyClone Laboratories, South Logan, UT) plus 1% ampicillin/streptomycin (Gibco). The authenticity of Vero E6 cells was verified through STR profiling by ATCC. The cells were tested negative for mycoplasma.

### Construction of chimeric SARS-CoV-2s with variant spikes

All spike mutations from variants were engineered into infectious cDNA clones of an early SARS-CoV-2 isolate, USA-WA1/2020, using a standard PCR-based mutagenesis method^22^. The full-length cDNAs of viral genomes containing the variant spike mutations were assembled by in vitro ligation. The resulting genome-length cDNAs served as templates for in vitro transcription of full-length viral RNAs. The full-length viral RNA transcripts were electroporated into Vero E6 cells. On day 2 post electroporation (when the electroporated cells developed cytopathic effects due to recombinant virus production and replication), the original viral stocks (P0) were harvested from culture medium. The P0 viruses were amplified on Vero E6 cells for another round to produce working viral stocks (P1). The infectious titers of the P1 viruses were quantified by plaque assay on Vero E6 cells^22^. The complete spike genes from the P1 viruses were sequenced to ensure no undesired mutations. The P1 viruses were used for neutralization tests.

### Characterization of wild-type and chimeric SARS-CoV-2’s with variant spikes

We quantified the P1 stocks for their genomic RNA content by RT-qPCR and for their infectious titers by plaque assay on Vero E6 cells. RNA copies of SARS-CoV-2 samples were detected by quantitative real-time RT-PCR assays using the iTaq SYBR Green One-Step Kit (Bio-Rad) on the LightCycler 480 system (Roche, Indianapolis, IN) following the manufacturer’s protocols. The absolute quantification of viral RNA was determined by a standard curve method using an RNA standard (in vitro transcribed 3480 bp containing genomic nucleotide positions 26,044 to 29,883 of SARS-CoV-2 genome)^5^. Infectious titers of SARS-CoV-2 samples were detected by plaque assay. Approximately 1.2 × 10^6^ Vero-E6 cells were seeded to each well of six-well plates and cultured at 37 °C, 5% CO_2_ for 16 h. Virus was serially diluted in DMEM with 2% FBS and 200 µl diluted viruses were transferred onto the monolayers. The viruses were incubated with the cells at 37 °C with 5% CO_2_ for 1 h. After the incubation, overlay medium was added to the infected cells per well. The overlay medium contained DMEM with 2% FBS, 1% penicillin/streptomycin, and 1% sea-plaque agarose (Lonza, Walkersville, MD). After a 2-day incubation, the plates were stained with neutral red (Sigma-Aldrich, St. Louis, MO) and plaques were counted on a lightbox. The detection limit of the plaque assay was 10 PFU/ml^23^. The ratio of viral RNA to PFU was calculated to indicate the specific infectivity of each virus.

### BTN162b2-vaccinated human sera

A panel of 20 serum samples were collected from 15 BNT162b2 vaccinees participating in the phase 1 portion of the ongoing phase 1/2/3 clinical trial (ClinicalTrials.gov identifier: NCT04368728)^6,14^. The participants provided written informed consent to take part in the study. The protocol and informed consent were approved by institutional review boards for each of the investigational centers participating in the study. The study was conducted in compliance with all International Council for Harmonisation Good Clinical Practice guidelines and the ethical principles of the Declaration of Helsinki. The sera were collected 2 or 4 weeks after two doses of 30 μg of BNT162b2 mRNA, spaced 3 weeks apart. As indicated in Supplementary Table 1, 5 of the 20 participants provided sera at both 2 and 4 weeks after the second dose of vaccine. The ages of human subjects are also presented in Supplementary Table 1. The serum donors were White, except for one donor who was Asian. All donors were of non-Hispanic/non-Latino ethnicity.

### Plaque-reduction neutralization test

A conventional 50% plaque-reduction neutralization test (PRNT_50_) was performed to measure the neutralizing titers of individual serum specimens^24^. The Vero E6 cells (1.2 × 10^6^ per well) were seeded to six-well plates. On the following day, individual sera were twofold serially diluted in culture medium with a starting dilution of 1:40. One hundred PFUs of WT or chimeric SARS-CoV-2 with variant spike were mixed with the serially diluted sera. After incubation at 37 °C for 1 h, the serum/virus mixtures were inoculated on to six-well plates with a monolayer of Vero E6 cells. After another 1 h incubation at 37 °C, 2 ml of 1% seaplaque agarose in DMEM containing 2% FBS and 1% P/S were added to the infected cells. Two days later, the plaques were stained by neutral red and counted on the white light board. The PRNT_50_ titer was defined the minimal serum dilution that suppressed >50% of viral plaques.

### Statistical analysis

Statistical analyses were performed by Graphpad Prism 9 for all experiments as detailed in legends to individual figures.

### Reporting summary

Further information on research design is available in the Nature Research Reporting Summary linked to this article.

## Supplementary information

Supplementary information

REPORTING SUMMARY

## Data Availability

Source data for generating the main figure are available in the online version of the paper. Any other information is available upon request.

## References

[1] Brown, C. M. et al. Outbreak of SARS-CoV-2 infections, including COVID-19 vaccine breakthrough infections, associated with large public gatherings — Barnstable County, Massachusetts, 2021. *Morbid. Mortal. Weekly Rep.* https://www.cdc.gov/mmwr/volumes/70/wr/mm7031e7032.htm?s_cid=mm7031e7032_w&fbclid=IwAR7032WV7036ul_A-l_VN_7015KX7034bedb7038CeLJKRwiDWZ-bIUuWmZMKbs7094xdhTiPLs (2021). 10.15585/mmwr.mm7031e2 PMC8367314 34351882

[2] Chen RE (2021). Resistance of SARS-CoV-2 variants to neutralization by monoclonal and serum-derived polyclonal antibodies. Nat. Med..

[3] Hou YJ (2020). SARS-CoV-2 D614G variant exhibits efficient replication ex vivo and transmission in vivo. Science.

[4] Liu Y (2022). The N501Y spike substitution enhances SARS-CoV-2 transmission. Nature.

[5] Plante JA (2021). Spike mutation D614G alters SARS-CoV-2 fitness. Nature.

[6] Polack FP (2020). Safety and efficacy of the BNT162b2 mRNA Covid-19 Vaccine. N. Engl. J. Med..

[7] Vogel AB (2021). BNT162b vaccines protect rhesus macaques from SARS-CoV-2. Nature.

[8] Abu-Raddad LJ, Chemaitelly H, Butt AA, National Study Group for, C.-V. (2021). Effectiveness of the BNT162b2 Covid-19 Vaccine against the B.1.1.7 and B.1.351 Variants. N. Engl. J. Med..

[9] Edara, V. V. et al. Infection and vaccine-induced neutralizing-antibody responses to the SARS-CoV-2 B.1.617 variants. *N. Engl. J. Med.*10.1056/NEJMc2107799 (2021). 10.1056/NEJMc2107799 PMC8279090 34233096

[10] Liu, J. et al. BNT162b2-elicited neutralization of B.1.617 and other SARS-CoV-2 variants. *Nature*, 10.1038/s41586-021-03693-y (2021). 10.1038/s41586-021-03693-y 34111888

[11] Liu Y (2021). Neutralizing Activity of BNT162b2-Elicited Serum. N. Engl. J. Med..

[12] Liu, Y. et al. BNT162b2-Elicited Neutralization against New SARS-CoV-2 Spike Variants. *N. Engl. J. Med.*10.1056/NEJMc2106083 (2021). 10.1056/NEJMc2106083 PMC8133696 33979486

[13] Planas, D. et al. Reduced sensitivity of SARS-CoV-2 variant Delta to antibody neutralization. *Nature*, 10.1038/s41586-021-03777-9 (2021). 10.1038/s41586-021-03777-9 34237773

[14] Walsh EE (2020). Safety and immunogenicity of two RNA-based Covid-19 vaccine candidates. N. Engl. J. Med..

[15] Thomas SJ (2021). Safety and efficacy of the BNT162b2 mRNA COVID-19 vaccine through 6 Months. N Engl J Med..

[16] Xie X (2021). Neutralization of SARS-CoV-2 spike 69/70 deletion, E484K and N501Y variants by BNT162b2 vaccine-elicited sera. Nat. Med..

[17] Falsey, A. R. et al. SARS-CoV-2 Neutralization with BNT162b2 Vaccine Dose 3. *N. Engl. J. Med.*, 10.1056/NEJMc2113468 (2021). 10.1056/NEJMc2113468 PMC8461567 34525276

[18] Liu, Y. et al. Delta spike P681R mutation enhances SARS-CoV-2 fitness over Alpha variant. Preprint at *BioRxiv*, 10.1101/2021.08.12.456173 (2021). 10.1016/j.celrep.2022.110829 PMC9050581 35550680

[19] Mlcochova, P. et al. SARS-CoV-2 B.1.617.2 Delta variant replication and immune evasion. *Nature*. **599**, 114–119 (2021). 10.1038/s41586-021-03944-y PMC8566220 34488225

[20] Li B (2022). Viral infection and transmission in a large, well-traced outbreak caused by the SARS-CoV-2 Delta variant. Nat. Commun..

[21] Steinhauer DA (1999). Role of hemagglutinin cleavage for the pathogenicity of influenza virus. Virology.

[22] Xie X (2020). An infectious cDNA clone of SARS-CoV-2. Cell Host Microbe.

[23] Xie X (2021). Engineering SARS-CoV-2 using a reverse genetic system. Nat. Protoc..

[24] Muruato AE (2020). A high-throughput neutralizing antibody assay for COVID-19 diagnosis and vaccine evaluation. Nat. Commun..

