# Supplementary material for: BNT162b2-elicited neutralization of Delta plus, Lambda, Mu, B.1.1.519, and Theta SARS-CoV-2 variants
Source: NPJ Vaccines. 2022 Apr 8;7:41. doi: 10.1038/s41541-022-00462-4 (PMC8993837; doi:10.1038/s41541-022-00462-4)
Supplement: Supplementary file 1 — Supplementary information [file 41541_2022_462_MOESM1_ESM.pdf]

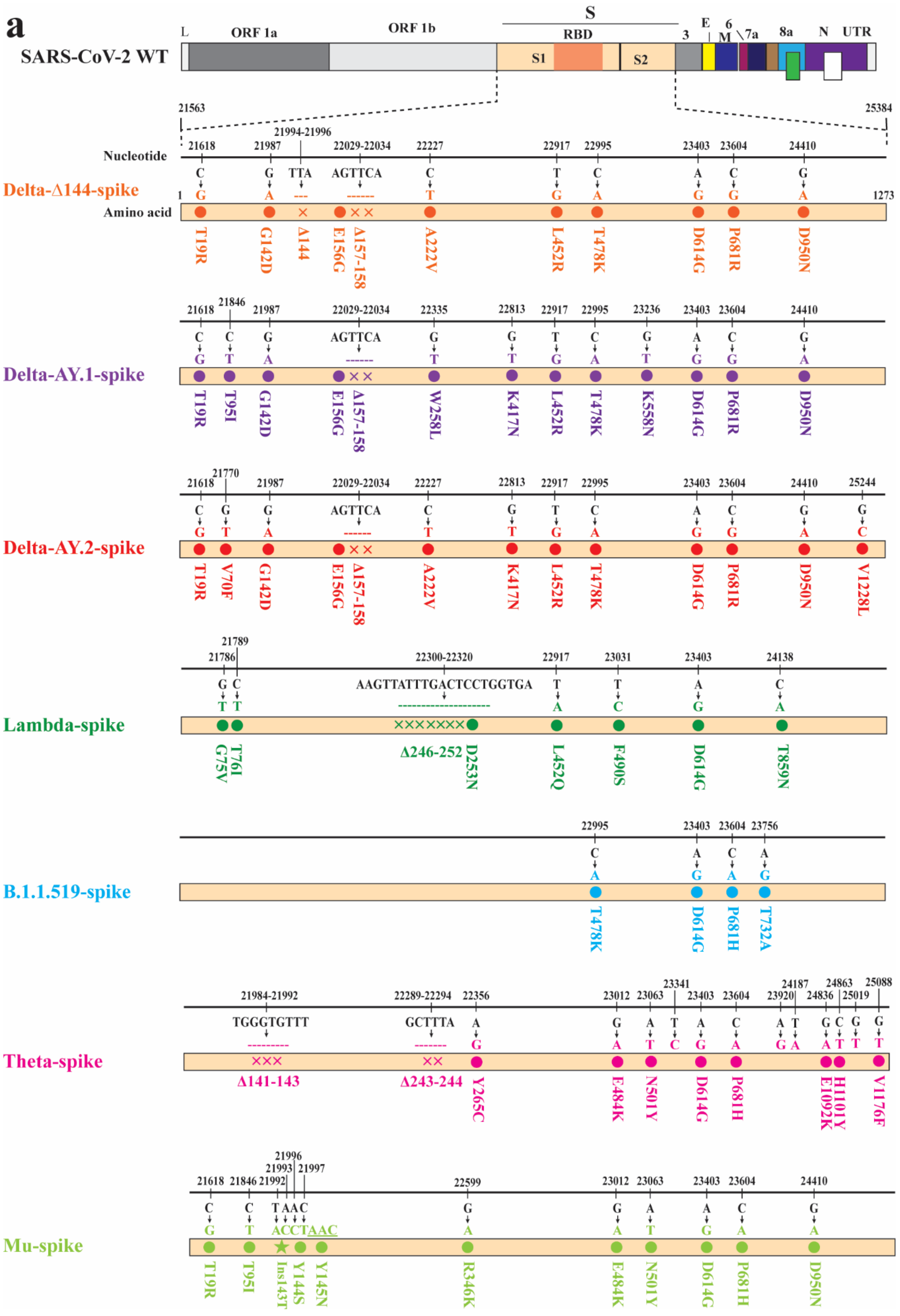

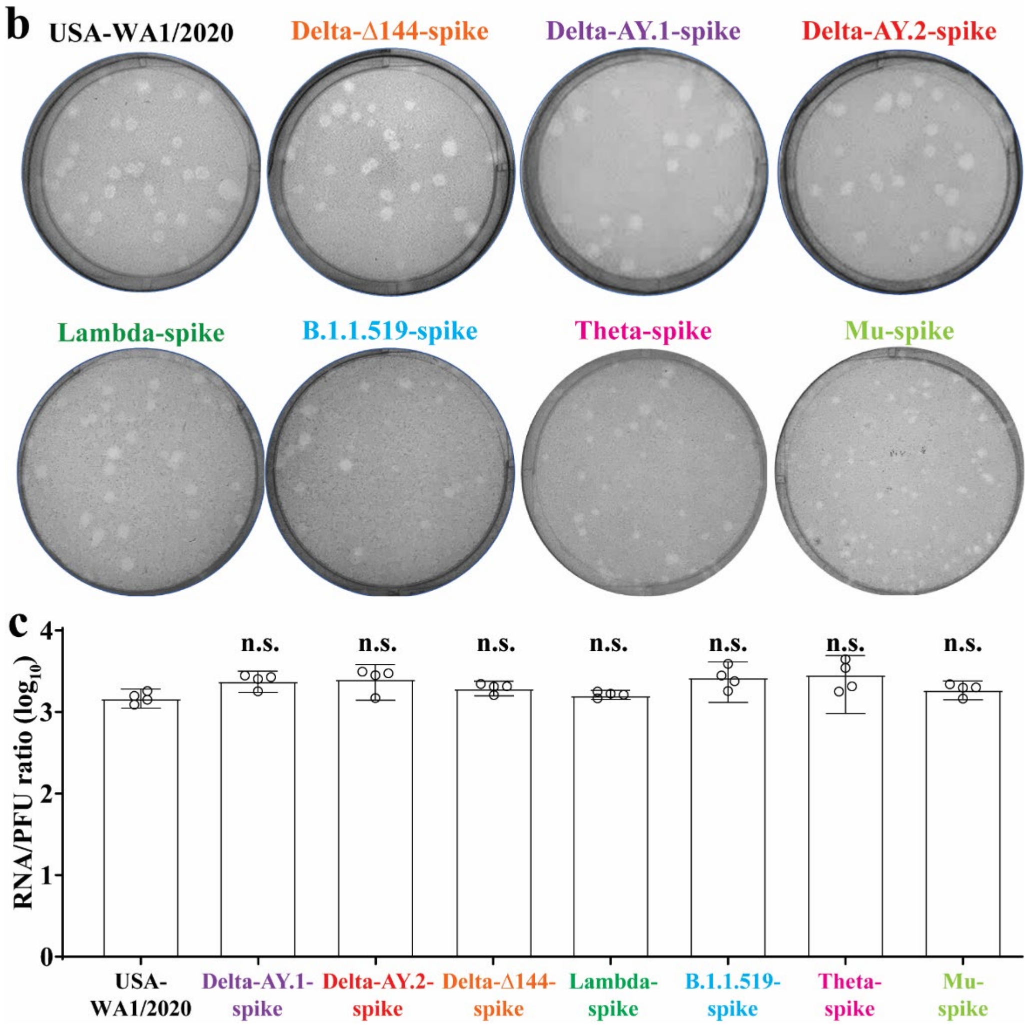

Supplementary Figure 1. Construction and characterization of SARS-CoV-2s with variant spikes.

a, Summary of engineered variant spike mutations. Mutations from variant spikes were engineered into USA-WA1/2020 SARS-CoV-2. Mutations and deletions are indicated by dots (●) and crosses (x), respectively. Nucleotide and amino acid positions are also shown. Regions of SARS-CoV-2 genome are indicated: L (leader sequence), ORF (open reading frame), RBD (receptor binding domain), S (spike glycoprotein), S1 (N-terminal furin cleavage fragment of S), S2 (C-terminal furin cleavage fragment of S), E (envelope protein), M (membrane protein), N (nucleoprotein), and UTR (non-translated region). **b,** Plaque morphologies of recombinant SARS-CoV-2s. Plaque assays were performed on Vero E6 cells in 6-well plates. **c,** Viral genomic RNA versus plaque-forming unit ratios (RNA/PFU) of recombinant SARS-CoV-2s. The genomic RNA content and PFU of individual virus stocks were measured by RT-qPCR and plaque assay, respectively. The RNA/PFU ratios were calculated to determine specific infectivities. All variants produced P1 virus stocks with infectious titers $>10^7$ PFU/ml. Dots represent individual biological replicates from 4 aliquots of viruses (n=4, one experiment). The values in the graph represent means with 95% confidence intervals. A non-parametric two-tailed Mann-Whitney test was used to determine significant differences between USA-WA1/2020 and variant viruses. *P* values were adjusted using the Bonferroni correction to account for multiple comparisons.

Differences were considered significant if $P < 0.05$ after correction; n.s. means no statistical difference.

Supplementary Table 1. PRNT$_{50}$ values of 20 sera from BNT162b2-vaccinated clinical trial participants against USA-WA1/2020 and SARS-CoV-2's with variant spikes

*†Serum			PRNT$_{50}$										
ID	Age	Week	&USA-WA1/2020				Delta-AY.1-spike	Delta-AY.2-spike	Delta-Δ144-spike	Lambda-spike	B.1.1.519-spike	Theta-spike	Mu-spike
			Exp1	Exp2	Exp3	GMT							
1	68	2	640	640	640	640	640	640	640	640	640	640	160
2	67	2	160	80	160	127	80	160	80	80	160	160	80
3	68	2	1280	640	640	806	1280	640	640	640	1280	640	640
4	65	2	320	640	320	403	320	320	320	640	640	320	160
5	30	2	320	320	320	320	80	320	320	320	320	320	320
6	23	2	640	320	640	508	320	160	320	640	640	320	320
7	54	2	1280	640	1280	1016	640	640	640	640	640	640	640
8	69	2	640	320	320	403	320	640	320	640	640	320	160
9	65	2	640	1280	640	806	640	640	640	1280	1280	640	640
10	38	2	320	640	320	403	640	640	640	640	640	640	160
11	44	2	640	640	640	640	320	320	640	640	640	1280	640
12	52	2	640	640	640	640	320	320	640	640	640	640	320
13	28	2	1280	1280	1280	1280	320	640	640	640	1280	320	320
14	69	4	320	640	320	403	320	320	160	640	640	320	160
15	68	4	320	320	320	320	320	640	640	640	640	320	160
16	26	4	320	320	320	320	160	160	640	640	640	320	320
17	54	4	640	640	640	640	640	640	640	1280	640	640	320

18	35	4	320	640	640	508	640	320	640	640	640	640	320
19	44	4	640	640	640	640	320	320	320	640	640	640	640
20	52	4	640	640	640	640	320	320	640	640	640	640	320
	[§]GMT		520	520	502	514	355	394	453	597	640	469	288
	[^]95% CI		401-674	393-688	394-639	406-651	258-489	311-500	346-592	462-771	519-790	375-585	217-383

*Five of the twenty participants donated pairs of sera at both 2 and 4 weeks after the second dose of vaccine. The paired sera have specimen IDs of 1 and 15, 7 and 17, 8 and 14, 11 and 19, and 12 and 20.

[†]The serum donors were White, except for donor 10, who was Asian. All donors were of non-Hispanic/non-Latino ethnicity.

[&]The data for USA-WA1/2020 are from three independent experiments. The results for other variants are from one experiment each. For each independent experiment, the individual $PRNT_{50}$ value is the geometric mean of duplicate plaque assay results; no differences were observed between the duplicate assays.

[§]GMT: Geometric mean neutralizing titers.

[^]95% CI: 95% confidence interval (95% CI) for the GMT.
